# Farmers’ Preferences Regarding the Design of Animal Welfare Programs: Insights from a Choice-Based Conjoint Study in Germany

**DOI:** 10.3390/ani11030704

**Published:** 2021-03-05

**Authors:** Iris Schröter, Marcus Mergenthaler

**Affiliations:** Faculty of Agriculture, South Westphalia University of Applied Sciences, Lübecker Ring 2, 59494 Soest, Germany; mergenthaler.marcus@fh-swf.de

**Keywords:** animal welfare, animal welfare measures, animal welfare programs, choice experiment, farmers’ preferences

## Abstract

**Simple Summary:**

Numerous animal welfare schemes have been developed aiming to improve animal welfare on a voluntary basis beyond legal regulations. The success of these schemes depends decisively on whether the design of these schemes is attractive to livestock farmers and, as a result, whether they are willing to participate and thus to implement the animal welfare measures regulated in these schemes. In this study, we investigated German livestock farmers’ preferences regarding the design of animal welfare schemes with a choice experiment. Farmers were asked to select their most preferred alternative among animal welfare schemes that differed in the specifications of the following four attributes: basis for remuneration (i.e., type of animal welfare measures to be implemented), commitment period, funding agency and compensation level. The basis for remuneration and the compensation level had the greatest influence on farmers’ decisions. The commitment period also affected farmers’ decisions. Independent of the livestock species kept, farmers preferred animal health as basis for remuneration, the higher compensation level and the longer commitment period. These findings could be incorporated into the development and refinement of animal welfare programs to make them more attractive to farmers and thus increase their willingness to participate.

**Abstract:**

As more animal welfare is required in livestock farming, several approaches have been developed to improve the well-being of farmed animals on a voluntary basis. Since farmers’ acceptance is important for the success of these approaches, their preferences should be considered when developing farm animal welfare programs. We used choice based conjoint analysis to investigate the preferences of 242 German livestock farmers (147 cattle farmers; 95 pig farmers) regarding the design of farm animal welfare programs. The conditional logit regression models show that the measures serving as basis for remuneration and the compensation level were of decisive importance for the farmers’ choices. The most preferred measure for assessing animal welfare, and thus as the basis for remuneration, was animal health. As expected, a higher compensation level led to greater acceptance of an animal welfare approach. The commitment period was only of subordinate importance with the longer commitment period being preferred. Our study outlines aspects of farm animal welfare programs that might encourage farmers to participate in these programs. Future programs could consider our findings by emphasising health parameters and by creating planning security through longer commitment periods and sufficiently high compensations for farmers’ efforts to improve animal welfare.

## 1. Introduction

Intensive livestock farming and its future orientation have been under discussion for years [1]. In addition to environmental effects of livestock production, this discussion focuses on improving farm animal welfare (FAW) standards. However, so far there is no agreement on how higher FAW standards can best be implemented on farms. One reason for this disagreement is that many stakeholders with partly conflicting interests are involved: the public, consumers, farmers, retailers, policy makers, researchers, and not to forget—the farm animals [2]. Another important reason could be the fact that FAW is a multidimensional construct that has not yet been fully understood [3].

In general, animal welfare can be seen as a state of physical and mental health arising from an animal’s ability to cope with its environment and from the subjective experiences and emotional assessments resulting from this adaption process [3]. While most stakeholders may agree with this definition, there does not yet exist a simple, single, generally accepted method to assess FAW. The multidimensionality of that theoretical construct makes it difficult to specify concrete measures for determining FAW and the way for data collection. This situation is further complicated by the fact that the importance attributed to different aspect of animal welfare may vary between different people even if they belong to the same stakeholder group, and, may also vary between animal species and individual animals [4,5,6]. 

These differing views on FAW are reflected by different legislation in different countries and by offering different FAW programs with a broad variety of different criteria. The programs primarily seek to improve public and consumer acceptance of animal husbandry practices [7,8,9]. It is, however, insufficient to consider only public or consumer acceptance when developing or refining FAW approaches. There are other stakeholders, whose acceptance strongly influences the success of FAW programs. In this context, it is important to highlight the role of farmers as key stakeholders, because they are the first link in the food supply chain [10,11,12,13]. They have to make the greatest contribution to the implementation of higher FAW standards. Therefore, as long as providing higher FAW standards is voluntary, it is plausible that attractive framework conditions within FAW programs could increase farmers’ willingness to participate in these programs. Unfortunately, the preferences of farmers regarding the design of FAW programs have rarely been investigated [14].

A useful method to better understand and predict farmers’ decisions about their participation in FAW programs is choice based conjoint analysis. This is a common method to determine preferences of individuals in choice situations, e.g., when deciding on a product or contract or other multi-attributive concepts [15]. Based on discrete choices considering all characteristics (attributes and attribute levels) of an object of investigation jointly, the extent to which the respective attribute levels contribute to the total utility of this object and how important the attributes are for preference formation can be calculated [16]. Choice experiments have repeatedly been applied to determine farmers’ preferences, e.g., for participating in agri-environmental schemes, for contract design or related to planting decisions [17,18,19,20,21]. However, we are aware only of two recent studies that used choice experiments to elicit farmers’ preferences related to the design of FAW programs. [14] applied this method to determine pig farmers’ preferences for the design of FAW approaches, [22] conducted a choice experiment with dairy farmers. Both studies contain only a few attributes of FAW programs and include very specific animal welfare measures (such as access to pasture 120 days per year [22] or providing three pieces of manipulable material [14]), thus leaving questions open. 

The present study contributes to the literature by examining farmers’ preferences for the design of FAW programs more comprehensively using choice based conjoint analysis. With regard to the animal welfare measures offered for choice, it includes categories of animal welfare measures to be able to examine preferences for superordinate types of measures rather than for individual measures. The focus of the study lies on the relevance of the characteristics of FAW programs, that means on the attributes and attribute levels, for farmers’ decisions regarding their participation in such schemes. Differences between farmers that might be attributed to the type of livestock kept are taken into account by segmentation, i.e., by running separate analyses for cattle and pig farmers.

The next section gives an overview of the literature on existing approaches for assessing and improving FAW. Furthermore, this section provides background information to understand the selection of the attributes and attribute levels for the choice experiment. The methodology section describes the framework conditions and the procedure of the choice experiment as well as the statistical analysis of the experiment. The results section contains descriptive statistics of the sample and presents the results of the choice experiment. In the discussion, we reflect on the results, relate them to the existing literature and point out the limitations of the study. Finally, we draw conclusions and outline further research needs. 

## 2. Literature and Background Information

In practice, FAW is assessed using three dimensions of measures [23]: Resource-based measures refer to the environment of the animals such as barn design, barn equipment, ventilation, light intensity or feed composition. Management-based measures focus on the procedures on the farm, for example, feeding routines, treatment of sick animals, regulations concerning mutilations, or documentation. Both, resource-based and management-based criteria are relatively easy to observe and to assess. However, they are rather prerequisites for good animal welfare, but they do not necessarily assess the actual state of physical or mental health of animals. The third dimension are animal-based measures. These measures rely on the condition and the behaviour of animals and are thus direct indicators of animal well-being. For example, a high level of abnormal behaviour indicates an animal’s inability to express natural behaviour; lameness scoring and recording of integument alterations provide information about physical health of an animal [4,23] as an aspect of animal welfare. Slaughterhouse reports about ante- and post-mortem findings can also serve as an information source on the health status of an animal. Even if the latter information can no longer improve well-being of the animal concerned, these data can support farmers to better assess and improve animal welfare on farm level [24]. 

Approaches aiming to improve FAW frequently rely on a combination of the different dimensions of measures described above. The specific measures included and their weighting depend on the initiators of the respective approach [23]. Legal regulations within the European Union (EU) relate primarily to resource-based and management-based criteria ensuring a minimum level of animal welfare [25,26]. However, parts of society call for animal welfare standards that go beyond the current legal regulations. In Germany legal regulations on FAW are partly laxer and partly stricter than those of the EU [27]. Irrespective of these demands and new scientific research on animal welfare, the chances of success within the EU as well as in Germany in further regulating FAW through legal requirements seem to be rather low [25,26,28]. Instead of this, policy makers emphasise the potential of market-driven approaches. These approaches should help consumers to choose products with an appropriate level of FAW through voluntary labelling, information provision and certification [26]. 

In Germany, several approaches seek to improve FAW on a voluntary basis. One program that is enjoying increasing popularity among farmers and consumers is the animal welfare initiative (Initiative Tierwohl) [29]. This cross-sector alliance of associations and companies from the agricultural, meat and food trade sectors aims to improve the well-being of pigs and poultry on a broad scale. Until 2020, food retailers participating in the initiative payed 6.25 cent per kg of pork and poultry meat and sausage sold into a fund. The money of the fund is used to compensate farmers who implement higher FAW standards for their additional costs [30]. Until 2018, the system worked as a kind of a private tax-and-subsidy system in the market, since there was no product segregation and no labelling of products at the point-of-sale [2]. Since 2019, food retailers affiliated to the “Initiative Tierwohl” have started labelling meat products with the four-level label “Haltungsform”, with the basic and mandatory requirements of the "Initiative Tierwohl" defining the second level of the label [31]. From 2021, the fund solution was partly replaced by market-based financing [30]. Additionally, there are various labels aiming to compensate the provision of more FAW through higher market prices for animal welfare products: One example is the label of the German animal protection association (Deutscher Tierschutzbund) that provides a basic and a premium level. The labels are awarded for pork and chicken meat as well as for eggs and milk [32]. Another example is the label of the registered association ‘Neuland e.V.’; it is awarded for pork, cattle, sheep and chicken meat as well as for eggs [33].

In addition, there are other animal species-specific approaches, which emphasise particular aspects of animal welfare, e.g., pasture-based milk programs or programs for the rearing of male layer chicks. An example is the label “PRO Weideland” of the PRO WEIDELAND Deutsche Weidecharta GmbH that primarily addresses aspects of pasture access of cattle [34]. Another example is the “BID Bruderhahn Initiative Deutschland e.V.” that focused on the rearing of male layer chicks and is currently being transferred into the “BID Brudertier Initiative Deutschland e.V.” with the aim of pushing the rearing of both sexes in all sectors of organic livestock farming and of solving further ethical problems in organic animal husbandry [35]. 

At federal level, the introduction of a national three-level governmental animal welfare label is planned; but is currently hampered due to political disagreements [36]. Finally, there are also regional programs aiming to improve FAW; the federal state Lower Saxony has subsidised pig farmers who abstain from tail-docking (“Ringelschwanzprämie”) since 2015 [37]. 

All the aforementioned approaches require animal welfare standards that go beyond legal regulations. Similar to legal regulations, they regulate predominantly management- and resource-based aspects, but to a lesser extent, they also address animal-based criteria. However, the extent to which the respective program contributes to higher FAW and challenges the farmers who have to implement the measures can vary considerably as every approach has its own criteria and levels [13]. Therefore, it seems reasonable to assume that farmers’ preferences for animal welfare programs depend on the type of measures included.

Besides preferences for the type of measures used to evaluate FAW, additional aspects may influence farmers’ willingness to participate in FAW programs. Farmers are, first and foremost, entrepreneurs. Therefore, financial incentives can be one of the most important motivations for providing higher FAW and accepting a specific FAW approach [1,12,13,38].

Furthermore, the initiator of FAW programs could be of interest. A study by [5] indicates that some farmers prefer commercial animal welfare approaches as they expect financial advantages, while other farmers favour strict and uniform governmental regulations to ensure good welfare of all farmed animals.

Investigations on farmers’ preferences in other areas of agricultural production show that the commitment period can also influence the attractiveness of a contract arrangement. These studies repeatedly point out that longer contracts terms are less attractive for farmers [17,19,20]. In contrast, other authors emphasise the advantages of long commitment periods, particularly in the context of long-term investments in FAW [39,40].

The following specific questions arise from the above:

1. Which type of measures do farmers prefer for assessing animal welfare within an FAW program?
2. Does the commitment period of FAW programs influence farmers’ decisions?
3. Do farmers prefer governmental or commercial approaches for providing higher FAW standards?
4. To what extend does the compensation level influence farmers’ decisions?

We intend to answer these questions using the choice experiment described in the next section. 

## 3. Methodology

### 3.1. The Choice Experiment

The choice experiment was conducted in summer 2018 as a part of a more comprehensive online survey on farm animal welfare of which other parts are used in [41,42]. The survey was intended to be answered only by livestock farmers. Therefore, the participants were motivated to participate through calls of professional farmers’ organisations and announcements in relevant agricultural magazines. Ten vouchers worth 25 Euro were raffled off to encourage participation in the survey. Prior to the activation, the survey was pretested with German livestock farmers to ensure understanding and clarity of the questions (cf. [41]).

Table 1 shows the animal welfare program attributes and attribute levels included in the choice stimuli. We derived the four attributes and their levels from qualitative interviews conducted at the end of 2015 (cf. [41]), supplemented by the recent literature, and from the German FAW approaches mentioned above. The attribute ‘object of remuneration’ defined the categories of FAW measures available for choice. The attribute levels involved all dimensions of measures for assessing animal welfare: environmental-based (housing system), animal-related (animal health and animal behaviour) and a bundle of environmental-based, management-based and animal-related (animal welfare criteria) measures. The rationale for the attribute levels was previous own research, which demonstrated that German livestock farmers consider animal health an important indicator of animal welfare, that they are open to new housing systems improving animal welfare [43], and that they also include animal behaviour when evaluating the well-being of their animals [41]. The naming of the attribute level “animal welfare criteria” was derived from the ITW’s naming of their FAW program measures (in German: ‘Tierwohlkriterien’) and describes a bundle of FAW measures which can be implemented within existing housing systems. The fact that this attribute level also contains aspects of the other attribute levels was deliberately accepted, since the emphasis here was on the combination of criteria from different dimensions. The attribute levels of the attribute ‘commitment period’ were determined against the background that implementing higher animal welfare standards represents a certain financial and organisational burden for farmers. Therefore, we chose two years as the minimum commitment period. This is one year less than the (maximum) commitment period of the German animal welfare approach ‘Initiative Tierwohl’. We offered a commitment period of 10 years as the second attribute level, since long-term planning security might be one aspect influencing the implementation of FAW measures [43]. To identify potential preferences for governmental or commercial regulation of FAW standards, these two alternatives were provided as attribute levels of the attribute ‘funding agency’. The two levels for the attribute ‘compensation level’ were derived from the pricing of the marketing company for ‘Neuland’ products that offers farmers participating in the program a fixed price for one year with a premium of 20–50 percent on the market price [44]. 

**Figure 1:**
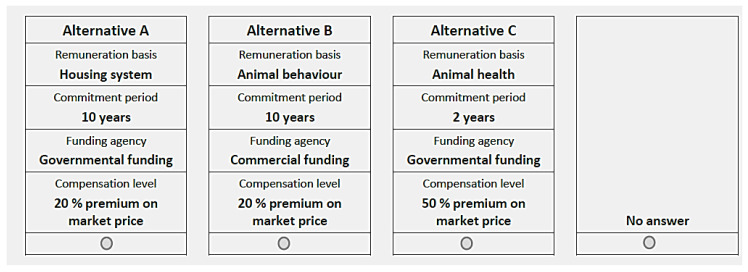
Example of a choice set.

We used the *orthoplan* procedure of SPSS to generate an orthogonal design, resulting in 32 choice-sets. In Figure 1, each choice set contained three choice stimuli and a none-option. Four choice sets were modified manually. They contained at least two identical stimuli and thus the duplicates did not provide any additional information [16]. Of the 32 choice sets, four randomly chosen sets were presented to each respondent. Consequently, every respondent had to choose four times among three hypothetical FAW programs and the none-option. The choice experiment was of the ‘unlabelled’ type, hence ‘Alternative A’, ‘Alternative B’ and ‘Alternative C’ in Figure 1 represent column assignments rather than alternative-specific labels.

### 3.2. Analysis of the Choice-Based Conjoint Experiment

Methodologically, the analysis of choice experiments is based on two theories [45]: the theory of [46] who argues that goods possess multiple characteristics (or attributes) and that these attributes generate utility and not the goods themselves; and the Random Utility Theory [47]. The random utility theory assumes that each individual maximizes his or her utility when choosing an alternative. 

We used the CLOGIT command of STATA, a conditional (fixed effects) logistic regression, to estimate the part-worth utilities of the attribute levels of the FAW approaches. According to this model, the probability $p_{km}$ that an individual chooses the alternative $c_{k}$ from a set $C_{m}$ of *m* alternatives ($c_{1}$,…,$c_{m}$) is a function of the utility *U* of alternative $c_{k}$ divided by a function of the utility of all alternatives in the set [48]:

$$p_{km}=p\left(c_{k}|C_{m}\right)=\;\frac{\exp\left(U\left(c_{k}\right)\right)}{\sum_{n=1}^{m}\exp\left(U\left(c_{n}\right)\right)}$$

The utility of each alternative is a linear function of the part-worth utilities $\beta_{ij}$ of the attributes:

$$U(c_{k})=\;\overset{q}{\underset{i=1}{\sum }}\;\overset{p_{i}}{\underset{j=1}{\sum }}x_{ij}.\;\beta_{ij}$$

The coefficient $\beta_{ij}$ is the part-worth utility of level *j* of attribute *i*. The dummy variable $x_{ij}$ indicates the presence ($x_{ij}=1)$ or the absence ($x_{ij}=0)$ of the level *j* of attribute *i* for alternative $c_{k}$. The coefficient $\beta_{ij}$ has to be estimated for each level of each attribute. One attribute level within each attribute serves as reference category for the other attribute levels; its part-worth utility is scaled to zero [49].

The importance $I_{i}$ of an attribute was derived from the range of the part-worth utilities of its attribute levels [16]:

$$I_{i}=\max\left\{\beta_{ij}\right\}-\min\{\beta_{ij}\}$$

The relative importance of an attribute $W_{i}$ was calculated by normalisation to ascertain its importance relative to other attributes [16,49]:

$$W_{i}=\;\frac{I_{i}}{\sum_{i=1}^{q}I_{i}\;}$$

Since various studies on FAW suggest that farmers are not a homogeneous target group [11,13,38] with the livestock species kept being an important factor influencing preferences regarding the characteristics of FAW measures [5,41,50,51], a-priory segmentation was performed. We assigned all types of cattle farmers to one segment and all types of pig farmers to the other segment. The segment of poultry farmers was excluded from the analysis due to the small number of those farmers in the sample. 

In addition, we excluded all participants who had chosen the opt-out option in all four choice tasks from the analysis of the choice experiment. We assumed that they did not want to answer or that they were overburdened with the task.

## 4. Results

### 4.1. Participants and Farm Characteristics

Of the 285 participants who had finalised the survey, 242 participants could be included in the analysis. Table 2 presents farm characteristics and demographic data for these remaining participants separately for the segment of cattle farmers (CF) and the segment of pig farmers (PF). 

The CF segment consisted of more than two-thirds dairy farmers, the PF segment mainly of sow keepers and pig fatteners. The number of participants for whom farming was the main source of income (full-time farms) did not differ between CF and PF [chi-square (1) = 0.79, *p* = 0.37]. The size of farmed area also did not differ between CF and PF [chi-square (3) = 2.39, *p* = 0.49]. Compared to Germany, the sample included an above-average number of full-time farms and farms with an above-average size of farmed land [52]. 

The average age of CF was similar to the average age of PF [t (235) = −0.71, *p* = 0.48]. The farmers in the present study were approximately as old as the farmers in the studies by [12,14] who report an average age of 45 and 43 years, respectively. 

Similar to the study by [12], who report more than 80 percent male participants, most of the participants in our study were male. However, significant differences between CF and PF existed [Chi-Square (1) = 13.93, *p* < 0.001)]. In the CF segment, there were about 20 percent more female respondents. 

The level of education of CF and PF was quite similar [Chi-Square (4) = 7.09, *p* = 0.13]. About half of the respondents were technicians in the field of agriculture and about one third of CF and PF hold an agriculture-related academic degree.

### 4.2. Choice Experiment: Estimation Results

**Figure 2:**
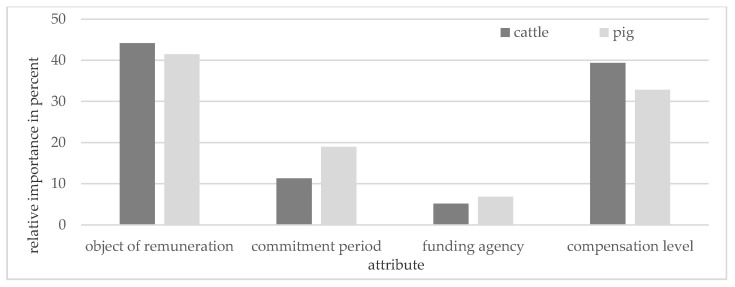
Relative importance of the evaluated farm animal welfare program attributes separately for cattle farmers and for pig farmers.

Figure 2 shows the relative importance of the evaluated FAW program attributes separately for CF and PF. The relative importance of the attributes followed the same order in both groups. The compensation level as offered in the choice experiment was not the most important attribute, neither for CF nor for PF; but CF gave higher importance to this attribute than PF. Both groups attached the highest relative importance to the object of remuneration, suggesting that this attribute had the highest impact on farmers’ decisions. The least relevant attribute for both CF and PF was the funding agency. 

Table 3 shows the estimation results of the conditional logistic regression models for CF and PF. The likelihood ratio tests indicate that the models are highly significant (*p* < 0.001). Therefore, the global null hypothesis that the attributes included in the models had no effect on farmers’ decisions regarding FAW approaches must be rejected. 

The coefficients represent the part-worth utilities of the attribute levels. As can be derived from the highest part-worth utilities within the attribute ‘object of remuneration’, both CF and PF found animal health criteria most desirable. The part-worth utilities for this attribute level differ significantly from the reference category ‘housing system’. Animal welfare criteria as the basis for remuneration were more appealing for CF compared to the reference category (*p* < 0.001) as well. In the PF model, the result is not so clear; but PF also tended to favour animal welfare criteria over the reference category (*p* = 0.052). PF were most critical about animal behaviour as object of remuneration; the part-worth utility of this attribute level is significantly lower than the part-worth utility of the reference category in the PF model. As the part-worth utility is also lower than those of the none-option, PF rather abstained from choosing an FAW program incorporating animal behaviour as basis for remuneration, unless the disadvantage was compensated by other preferred program components, such as a high price premium. In contrast, CF were more likely to choose one of the proposed FAW approaches than the none-option, regardless of the combination of attribute levels.

A longer commitment period was preferred by PF and, in tendency, by CF. In contrast, whether commercial or governmental funding was offered neither affected the decisions of CF nor PF.

As expected, a price premium of 50 percent on the market price has a significantly higher part-worth utility for CF and PF compared to a price premium of 20 percent. 

## 5. Discussion

We investigated German famers’ preferences regarding the design of FAW programs using choice based conjoint analysis. The results show that three out of four attributes included in the choice experiment influenced farmers’ preferences. Regardless of the livestock species kept, the most important attribute for the farmers’ choice was the object of remuneration, with animal health being the most preferred attribute level. Compensation level was only slightly less important for farmers’ choice than the object of remuneration and ranked second. As expected, a higher price premium increased the probability of choosing an alternative. Commitment period was less relevant; nonetheless, the longer commitment period of ten years was preferred over the short commitment period of two years. It did not affect the decisions of either cattle or pig farmers whether governmental or commercial funding was offered for choice. 

However, to be able to better assess the results, some limitations of the study are worth noting. 

First, when interpreting the results, it is important to keep in mind that the relative importance of the attributes depends on the attribute levels included. In particular, the relative importance of the attribute ‘compensation level’ is very dependent on the range of compensation offered in the choice experiment. If other compensation levels had been included, the results would most likely have been different.

Second, the attribute levels of the attribute ‘object of remuneration’ included in the study are rather abstract. The advantage of this approach is that it allowed us to include all farmers in a single survey, regardless of the livestock species kept. In this way, we were able to determine the farmers’ general acceptance of different types of FAW measures and also to compare cattle and pig farmers. The disadvantage is that it implied a certain imprecision since the concrete design of the measures (e.g., whether the housing system is a barn with or without outdoor climate area) was left to the implicit pre-assumptions of the farmers.

Third, we used a conditional logit model. The underlying behavioural model of conditional logit models assumes that the basis on which individuals makes choices are the characteristics of the alternatives. That means that in conditional logit models a choice among alternatives is treated as a function of the characteristics of the alternatives, rather than the characteristics of the individual making the choice [53]. The latter aspect also points to the limitations of conditional logit models and thus of our analysis: heterogeneity among farmers that might arise from individual characteristics, such as age, livestock units, attitudes towards animal welfare labels or participation in quality assurance schemes [14,22] was not considered.

Finally, the empirical analysis is based on an ad hoc sample that is not representative for the population of German farmers. That means that the conclusions drawn must be interpreted with caution. 

Despite shortcomings, we aim to draw some conclusions discussed in more detail below. As expected, the compensation level strongly influenced farmers’ choice, which is in accordance with several previous studies emphasising the importance of monetary incentives as a motivation for participating in FAW programs [1,12,13,14,22,38,54]. However, an essential finding of our research is that the compensation level was not the most important attribute. As addressed earlier, the result is not independent of the attribute levels included in the choice experiment. The compensation level probably would have been most important if the range between the lower and the upper level would have been greater. Nevertheless, the result is surprising, since the difference between the 20 and 50 percent price premium offered for choice was already substantial.

The major importance of the object of remuneration might be explained, on the one hand, by the fact that farmers might have considered that the implementation of higher FAW standards puts a certain organisational and financial burden on them. This burden depends to a large extend on the measures that serve as the basis for assessing FAW and thus for remuneration, as these measures determine the adjustments of the production system, the production processes and the management to be made. The perceived ability to deal with these challenges decisively influence farmers’ acceptance of FAW approaches [54,55]. At this point it is important to note that the effort, i.e., labour and/or money, required to implement FAW measures, could be an important factor in the failure of voluntary FAW programs because the more effort and resources required to comply with a FAW program, the less farmers are willing to participate [14]. On the other hand, this attribute might have provided information on non-use values of the FAW programs and thus have been important for farmers’ decisions. Non-use values with regard to FAW refer to the value that farmers derive from economic goods related to the well-being of the livestock independent of any direct use, present or future, the farmer might make of the animals [23]. A relevant type of non-use values refers to the self-perception of farmers: Several studies show, that farmers are more satisfied and enjoying their work if they feel their animals are well treated and healthy [22,23,56,57]. Results of a study by [56] indicate that nonmonetary factors related to internal esteem and taking pleasure in healthy farm animals can be as motivating to improve animal welfare as monetary factors. 

This high non-monetary appreciation of animal health, together with the consideration that a high level of animal health can also increase animal performance and thus productivity [58,59] may have led to this attribute level being preferred as the basis for remuneration by both cattle and pig farmers. Furthermore, German farmers are already accustomed to providing animal health data to some institutions due to legal requirements and participation in quality programs. The provision of (additional) animal health data within the framework of an FAW approach might therefore appear to be easy to implement since poor animal health caused by disease, injury or deformity is often straightforward to recognise and can be quantified relatively objectively and reliably [60]. In addition, farmers might perceive animal health as the measure with the lowest investment costs for implementation as important health issues are rather linked to management practices and less related to infrastructural adjustments [61,62].

In this context it should be noted that animal health alone is not sufficient for assessing animal welfare–at least not if animal health is understood only as the absence of clinical signs or injuries [63]. Animal health might then be the stand-alone indicator of animal welfare, if the WHO definition of human health as ‘a state of complete mental, physical and social well-being and not merely freedom from disease and infirmity’ [64] would be applied to farmed animals. This definition of health is very close to the definition of animal welfare by [3] mentioned in the introduction of this paper. For determining states of this ‘extended’ animal health, further parameters would then have to be captured in addition to the health parameters currently recorded, in particular animal health measures that allow conclusions to be drawn about ‘invisible’ health parameters like the stress level of the animals (e.g., by periodically measuring glucocorticoid levels) [63]. However, whether farmers would then still prefer animal health as basis for remuneration would have to be subject of further research.

The high appreciation of animal welfare criteria as object of remuneration, understood as a bundle of different adaptive measures within existing housing and infrastructure systems for assessing FAW, might be explained by the farmers’ familiarity and acceptance of animal welfare programs that follow such an approach. In particular, the expectation that these programs would increase consumer acceptance may have contributed to this result [1]. A remuneration based on the design of the animal housing systems was less preferred, probably due to the expectation of high adjustment costs. This assumption is supported by the fact that farmers often doubt that investments in higher FAW standards will pay off and fear that animal welfare products will remain a niche market [1,13]. The results regarding animal behaviour as the basis for remuneration were more differentiated. Even though some recent studies show that farmers have a comprehensive understanding of FAW, including animal behaviour [10,65], the pig farmers in this sample seem to regard this a rather inappropriate basis for remuneration. This assumption is supported by a study by [38] who report that appropriate animal behaviour for assessing FAW is rather unimportant for pig farmers. A study by [55] confirms the differences between cattle and pig farmers found in the present study. The authors describe that for cattle farmers the animals’ opportunity to engage in natural innate behaviour is considerably more important than for pig farmers. Possibly, pig farmers generally consider the recording of animal behaviour to be rather ineffective in improving animal welfare and fear a high additional workload. Although some commercial solutions for automated recording of pig behaviour are already available, there is still a great need for research and development in this regard and for convincing farmers of the benefits of these technologies [66,67]. Cattle farmers might be more relaxed about recording animal behaviour, as systems for automatic recording (e.g., activity, rumination) are already widely used in dairy farming (e.g., [68,69]).

Even if the commitment period was of less importance, the respondents preferred the long period of ten years. This result contrasts with studies on contract design in relation to other aspects of agricultural production, which report that farmers favour considerable shorter contract periods [17,19,20]. However, this long contract term might have been interesting for the farmers of this study, as it would provide long-term planning security regarding the requirements to be met and the remuneration [40]. A study by [39] also point to the fact that the implementation of higher FAW standards often require considerable investments that farmers will only make if these investments are covered by increased revenues in the long term. However, due to the studies cited above that identified short-term contracts as more attractive to farmers, it could be beneficial to offer both short and longer terms in FAW programs, as some farmers may be reluctant to commit to a long-term program.

In apparent contradiction to this need of security is the finding that the funding agency was irrelevant to the farmers’ decision. This is astonishing as farmers are particularly distrustful of other supply chain actors. On the other hand, they believe that these actors could play an important role in stimulating animal friendly production, because of their market power [70]. However, farmers are also increasingly losing confidence in policies. Due to constantly changing parameter of agricultural policies, they feel extremely insecure about the future of their farms. They complain about the excessive number of legal regulations that make adaptions more and more difficult and about a lack of perspectives and planning uncertainty due to unclear political plans for the future [71]. Furthermore, government funding could make farmers feel degraded to petitioners [72]. One of the farmers, who left a message in the closing question of the survey, summarised it as follows: *‘No subsidies for any programs, but fair prices for honest work’*.

Finally, this statement leads to the question of what prices can be considered to be ‘fair’ for which FAW aspects and who should pay for improved FAW. Although other stakeholders’ views, e.g., the views of citizens or consumers, were not subject of this study, it is worth noting that their preferences for FAW programs may differ from those of farmers and that there may be discordance in their perception of the level of FAW offered [14,73]. For large-scale success of FAW programs, however, it seems important to develop a common understanding of FAW among different stakeholders. Presumably, developing this common understanding will be an ongoing process in which farmers’ preferences will need to be considered along with other stakeholders.

## 6. Conclusions

An appropriate design of FAW programs is crucial to transfer farmers’ theoretical willingness to improve animal welfare into practice. Our study clearly indicates that the compensation level and the basis for remuneration have an outstanding importance for farmers’ decisions to participate in particular FAW programs. Regardless of the type of livestock kept, farmers of our study preferred animal health as basis for remuneration for efforts to improve animal welfare. Thus, FAW programs could put more emphasis on measures related to animal health criteria to assess animal welfare. For this purpose, more of these criteria might be included in FAW programs, in particular animal health measures that would allow conclusions to be drawn about ‘invisible’ health parameters, thereby allowing a more comprehensive assessment of animal welfare. In this context, it would be necessary to define appropriate parameters and to generate reliable, cost- and time efficient approaches to obtain these data. The development of cost-effective devices allowing automatically capturing and analysing aspects of animal behaviour that are relevant for assessing FAW, particularly in pig farming, could lead to higher acceptance of measures related directly to animal behaviour. In addition, more attention should be paid to the farmers’ desire for long-term planning security by providing long-term commitment periods within FAW programs.

Future investigations should aim to gain more knowledge about farmers’ preferences regarding the basis of remuneration in FAW approaches by mentioning concrete measures for assessing FAW. The measures presented for choice need to be adapted to the respective livestock species, but should also allow an overall assessment of farmers’ preferences, independent of the livestock species kept. Including additional attribute levels of the other attributes could provide information on preferred FAW program options that is more detailed. As consistent standards within the European Union are a prerequisite for competitive neutrality among farmers, future studies should be carried out not only at national but also at European level.

## Figures and Tables

**Table 1:** Attributes and attribute levels of the choice-based conjoint study.

Attribute	Number of Attribute Levels	Description of Attribute Levels
**Object of remuneration**	4	Animal welfare criteria
		Animal health
		Animal behaviour
		Housing system
**Commitment period**	2	ten years
		two years
**Funding agency**	2	Government
		Commercial
**Compensation level**	2	50% price premium on market price
		20% price premium on market price

**Table 2:** Characteristics of the farms and sociodemographic data of the farmers, separately for cattle farmers (N = 147) and pig farmers (N = 95).

	Cattle Farmers		Pig Farmers	
	N	Percent ^1^	N	Percent ^1^
**Farm characteristics**				
Relevance as source of income				
Main source of income	118	80.27	81	85.26
Sideline	28	19.05	14	14.74
Missing	1	0.68		
Farmed area				
less than 50 ha	33	22.45	28	29.47
50 to less than 100 ha	51	34.69	34	35.79
100 to less than 200 ha	38	25.85	19	20.00
200 ha and more	25	17.01	13	13.68
Missing			1	1.05
Main production branch				
Dairy cattle	106	72.11		
Suckler cows	18	12.24		
Beef cattle	23	15.65		
Sow keeping			41	43.16
Piglet rearing			3	3.16
Pig fattening			51	53.68
**Characteristics of the farmers**				
Age ^2^ [mean (SD)]	144	44.12 (14.08)	93	45.39 (12.18)
Gender				
Female	41	28.28	8	8.42
Male	104	71.72	87	91.58
Missing	2	1.36		
Education level				
Apprenticeship in agriculture	12	8.16	5	5.26
Technicians (agriculture)	62	42.18	50	52.63
Academic degree (agriculture)	47	31.97	32	33.68
Still in education/unrelated education	17	11.56	3	3.16
Missing	9	6.12	5	5.26

^1^ otherwise stated; ^2^ age: three missing values for cattle farmers and two missing values for pig farmers.

**Table 3:** Results of the conditional logistic regression models for cattle farmers and pig farmers. The coefficients (Coef.) represent the part-worth utilities of the individual parameters.

	Cattle Farmers				Pig and Poultry Farmers			
Test statistics	Observations		2 352		Observations		1 520	
	Log likelihood		−718.39		Log likelihood		−466.58	
	Chi2		193.50		Chi2		120.43	
	*p*-value		<0.001		*p*-value		<0.001	
	Mc Fadden’s R^2^		0.12		Mc Fadden’s R^2^		0.11	
Parameter	Coef.	95 % CI		*p*-value	Coef.	95 % CI		*p*-value
Object of remuneration								
Animal welfare criteria	0.55	0.27	0.83	<0.001	0.36	0.00	0.73	0.052
Animal health	0.72	0.43	1.02	<0.001	0.57	0.20	0.93	0.003
Animal behaviour	0.10	−0.21	0.42	0.532	−0.54	−0.96	−0.12	0.012
Housing system	0.00				0.00			
Commitment period								
Ten years	0.18	−0.02	0.39	0.077	0.51	0.24	0.77	<0.001
Two years	0.00				0.00			
Funding agency								
Government	0.08	−0.12	0.29	0.411	−0.18	−0.45	0.09	0.183
Commercial	0.00				0.00			
Compensation level								
50 % price premium	0.64	0.44	0.85	<0.001	0.88	0.60	1.15	<0.001
20 % price premium	0.00				0.00			
None	−0.55	−0.96	−0.14	0.009	−0.04	−0.51	0.44	0.883

## Data Availability

The data presented in this study are available on request from the corresponding author. The data are not publicly available due to our university’s privacy policy.
